# Supplementary figures and images for: Expanding the Drosophila toolkit for dual control of gene expression
Source: eLife. 2024 Apr 3;12:RP94073. doi: 10.7554/eLife.94073 (PMC10990484; doi:10.7554/eLife.94073)

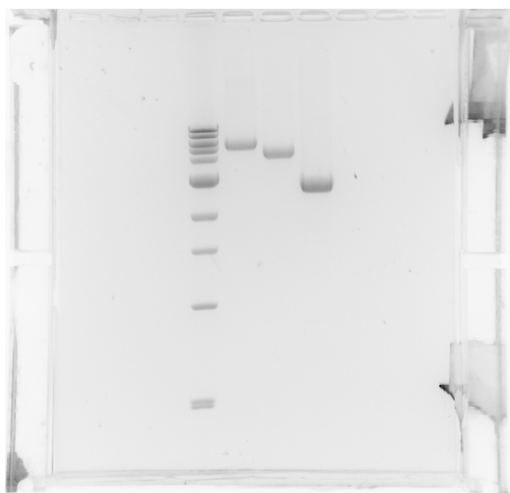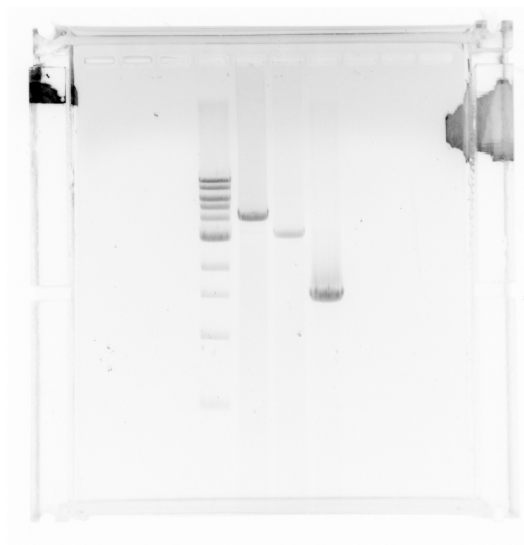

Supplement: Figure 5—source data 1. [file elife-94073-fig5-data1.zip › Figure 5-source data 1/Figure 5-source data 1.pdf]

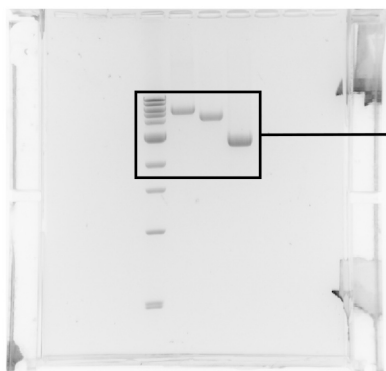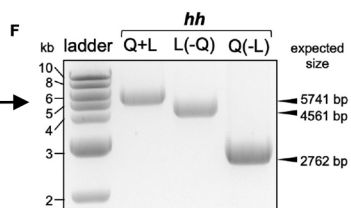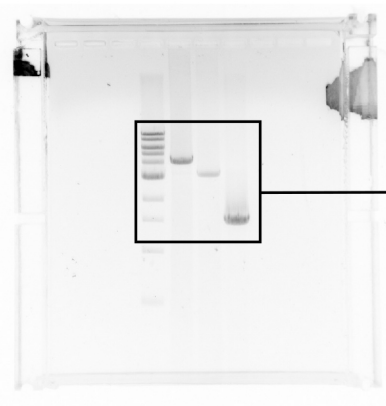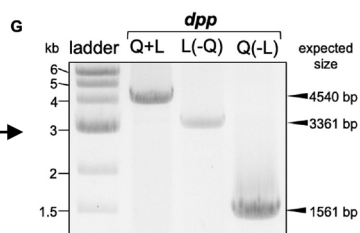

Supplement: Figure 5—source data 2. [file elife-94073-fig5-data2.zip › Figure 5- source data 2/Figure 5-source data 2.pdf]
